# Development of a predictive model using automated machine learning for Carbapenem-resistant Organisms (CRO) infections in hospitalized patients

**DOI:** 10.3389/fmicb.2026.1775344

**Published:** 2026-04-01

**Authors:** Dong Fang, Rui Shi

**Affiliations:** 1 Department of Cardiovascular Medicine, CR and WISCO General Hospital, Hubei, China; 2 Department of Pharmacy, CR and WISCO General Hospital, Hubei, China

**Keywords:** Carbapenem-resistant Organisms (CRO), clinical decision support, improved hannibal barcid optimization algorithm, machine learning, predictive model, risk stratification

## Abstract

**Objective:**

This study aimed to develop a predictive model based on Automated Machine Learning (AutoML) to achieve early and precise warning of Carbapenem-resistant Organisms (CRO) infections in hospitalized patients, thereby providing technological support for proactive prevention and control.

**Methods:**

A retrospective cohort study design was employed. Data from 958 hospitalized patients with CRO and Carbapenem-susceptible Organisms (CSO) infections were collected at CR and WISCO General Hospital between January 2022 and June 2025. An AutoML framework based on an Improved Hannibal Barcid Optimization algorithm (IHBO) was proposed. The model was constructed through dual-stage optimization (feature selection and hyperparameter tuning) and compared against five traditional algorithms, including Logistic Regression (LR) and Support Vector Machine (SVM). Model performance was evaluated using metrics including the Area Under the Receiver Operating Characteristic Curve (AUC), the Area Under the Precision-Recall Curve (AUPRC), and the F1 score. Key feature contributions and interaction mechanisms were analyzed using Shapley Additive exPlanations (SHAP).

**Results:**

Regarding model performance, the IHBO-optimized AutoML model demonstrated superior discriminative ability and robustness in the independent test set compared to others. Its ROC-AUC reached 0.8941 and PR-AUC reached 0.0.8844, significantly higher than those of the other models. Simultaneously, it achieved an F1 score of 0.8114, with sensitivity and specificity of 0.7917 and 0.8392, respectively. Calibration analysis indicated this model had the highest accuracy in predicted probabilities (Brier Score = 0.134). Decision curve analysis confirmed its significant clinical net benefit across the 1–71% risk threshold range. Feature analysis identified five core predictors ranked by importance: Antifungal Medication Usage, ICU Length of Stay Stratification, APACHE II Score > 15, Aminoglycoside Antibiotic Exposure, and Indwelling Urinary Catheter Use. SHAP interaction analysis further revealed: (1) Antifungal use significantly increases CRO risk, especially in patients with APACHE II > 15; (2) ICU stay duration shows dose-response relationship with CRO risk, amplified when APACHE II > 15; and (3) Combined use of aminoglycoside and urinary catheter creates synergistic risk, indicating additive effect of multiple risk factors; (4) Triple high-risk combination (antifungal + ICU > 7 days + APACHE II > 15) shows highest SHAP values, requiring intensive infection control measures. Based on these findings, a clinical decision support tool integrating the key features was developed, enabling the visual output of individualized infection risk.

**Conclusion:**

The IHBO-AutoML model developed in this study overcomes the limitations of traditional static methods. It employs explainable machine learning to elucidate the core driver mechanism involving the synergy between antifungal use and critical illness, the biological feedback loop linking prolonged ICU stay and organ failure, and the spatially specific resistance evolution induced by the interaction of drugs and medical devices. The model provides a precise tool for proactive prevention and control, facilitating the transition in carbapenem-resistant organism management modes.

## 1 Introduction

The prevention and control of drug-resistant bacterial infections has become a critical challenge for global public health systems. In recent years, alongside the widespread clinical application of broad-spectrum antibiotics, the infection rate of Carbapenem-resistant Organisms (CRO) has shown a significant upward trend (De Pascale et al., 2025; Falcone et al., 2023). Relevant studies in Germany revealed (Wilke et al., 2022) that the mortality rate of patients infected with such multi-drug resistant bacteria is more than three times higher than those infected with susceptible strains, with an annual increase in direct medical costs exceeding $4 billion. This poses a systemic threat to the sustainable operation of healthcare systems. Particularly noteworthy is that hospitalized patients, due to the combined factors of compromised immune barriers, frequent invasive procedures, and extensive antibiotic exposure, have become an ultra-high-risk group for CRO infections (Heo, 2021; Zhou et al., 2024; Bai et al., 2025). Traditional infection surveillance primarily relies on microbiological culture results and clinical judgment, suffering from limitations such as significant reaction lag (typically 48–72 h delay) and high rates of missed diagnoses, making early precise intervention difficult to achieve (Tabah et al., 2023; Righi et al., 2023). This passive prevention and control model sharply contradicts the modern infection management concept of “front-shifting intervention points,” urgently necessitating the development of proactive early warning technologies to break through the current bottleneck in prevention and control.

The rapid advancement of machine learning technology offers a revolutionary pathway to address the aforementioned challenges. Compared to traditional statistical models, machine learning algorithms demonstrate three unique advantages in complex healthcare data scenarios (Castellanos et al., 2024; Tuinte et al., 2025; Theodosiou and Read, 2023; Aiken et al., 2021): Firstly, their nonlinear modeling capabilities can effectively capture potential interaction effects among multi-dimensional risk factors, avoiding the cognitive limitations associated with manually predefining relationship patterns. Secondly, their adaptive feature learning mechanisms can automatically identify discriminant patterns within high-dimensional data, significantly enhancing the detection sensitivity for subtle warning signs. Thirdly, algorithms based on ensemble learning frameworks can strengthen model robustness through bias-variance tradeoff optimization strategies, maintaining stable predictive performance in noisy environments. Existing research has confirmed (Bonazzetti et al., 2025; B et al., 2025) that machine learning achieves 20–35% higher predictive performance compared to traditional scoring systems in scenarios such as sepsis early warning and nosocomial infection risk stratification, signifying that intelligent prediction technology is gradually reshaping the paradigm of infection prevention and control. Despite the significant progress in existing research, current clinical prediction models still face three core challenges (Muhsal et al., 2025; Yuan et al., 2025; Pan et al., 2025): First, the variable selection process often relies heavily on manual prior knowledge, making models susceptible to the subjective cognitive biases of the researcher. Second, most algorithms carry overfitting risks when handling high-dimensional, small-sample-size medical data, exhibiting significant performance degradation during cross-center validation. Finally, the “black-box” nature of mainstream deep learning models severely hinders clinical interpretability, making it difficult to meet the rigid demand for logical transparency in medical decision-making. These issues are particularly prominent in the field of CRO prediction—where the heterogeneity of microbiological data, the complexity of underlying patient comorbidities, and the dynamic nature of treatment trajectories collectively constitute a multidimensional spatiotemporal interleaved high-order nonlinear system, posing difficulties for accurate modeling using traditional machine learning architectures.

This study addresses the above scientific problems, aiming to construct an innovative machine learning framework for early intelligent warning of CRO infections in hospitalized patients. By integrating adaptive risk identification technology with interpretability enhancement mechanisms, the research focuses on overcoming key bottlenecks in modeling high-dimensional medical data, including feature selection optimization, enhancement of model generalizability, and visualization of decision logic. It seeks to provide a methodological foundation for establishing a precise and actionable infection early warning system. The anticipated outcomes are expected to catalyze the shift in multidrug-resistant organism prevention and control from passive response to active defense, thereby boosting the development of smart healthcare.

## 2 Materials and methods

### 2.1 Study population and data collection

This retrospective cohort study enrolled hospitalized patients with laboratory-confirmed Carbapenem-resistant Organisms (CRO) or Carbapenem-susceptible Organisms (CSO) infections at CR & WISCO General Hospital between January 2022 and June 2025. The study population comprised 958 hospitalized patients with laboratory-confirmed infections caused by either Carbapenem-resistant Organisms (CRO, *n* = 480) or Carbapenem-susceptible Organisms (CSO, defined exclusively as patients with culture-confirmed infections caused by carbapenem-susceptible Gram-negative bacteria, *n* = 478). This design establishes that the predictive model specifically identifies carbapenem resistance among existing infections rather than predicting de novo infection risk. The ethics committee of CR & WISCO General Hospital granted exemption from informed consent (Approval No.: CRWG2025R058J) in accordance with the Declaration of Helsinki, considering the retrospective nature of data collection.

Inclusion criteria: (1) fulfillment of diagnostic standards referenced in the Hospital Infection Diagnostic Criteria (Vincent, 2002); (2) restriction to the first infection episode in cases of recurrent infections; and (3) microbiological confirmation of all isolates through standardized identification and susceptibility testing.

Exclusion criteria: (1) incomplete clinical documentation; (2) Removes Gram-negative bacilli infections detected within 48 h of admission — ensures that this study exclusively addresses hospital-acquired (nosocomial) infections, as per standard epidemiological definitions for healthcare-associated infections requiring ≥ 48 h of hospitalization prior to onset; (3) confirmed specimen contamination; or (4) discharge, mortality, or treatment discontinuation within 48 h of admission.

Data abstraction employed structured collection forms to extract variables from the electronic medical record system. Dual verification by certified researchers ensured data integrity. Captured features spanned six domains: Demographic characteristics (age, sex); comorbidities (hypertension, diabetes mellitus, coronary artery disease, cerebrovascular disease, chronic kidney disease, malignancy, immunosuppression); infection/treatment parameters [specimen source, hospitalization > 20 days, ICU admission with stratified length-of-stay (0, 1–7, > 7 days), surgical interventions, invasive procedures (mechanical ventilation, central venous/arterial catheterization, indwelling urinary catheter), and vasoactive medication use]; antimicrobial exposure [carbapenems, aminoglycosides, glycopeptides (vancomycin/teicoplanin), fluoroquinolones, third-generation cephalosporins, β-lactam/β-lactamase inhibitor combinations (e.g., piperacillin-tazobactam, penicillins, and antifungals (e.g., fluconazole), with duration > 7 days]; severity scores (APACHE II, qSOFA, Charlson Comorbidity Index); and the primary outcome of laboratory-confirmed CRO infection during hospitalization. Time-zero was defined as the timestamp of initial clinical suspicion triggering laboratory testing for CRO infection (typically ≥ 24 h before confirmation). All predictors were stringently censored at this pre-diagnosis time-zero to eliminate look-ahead bias and reverse causality risks. Specifically: (i) Features including “Hospitalization > 20 days” used only pre-suspicion data; (ii) ICU admission duration was truncated at clinical suspicion time; (iii) Antimicrobial exposure windows ended 24 h before suspicion. EHR timestamp audits confirmed temporal validity (e.g., no hospitalization days accumulated after suspicion).

Before model development, the completeness of the analytical dataset (*n* = 958) was assessed. A missingness analysis revealed that all variables had a missing rate of < 5%, with most key predictors (e.g., APACHE II score, antimicrobial exposure history) missing in < 2% of cases. The detailed missing percentage for each variable is provided in Supplementary Table S1. To ensure prevention of information leakage, all imputation and standardization procedures were performed within each cross-validation fold exclusively, rather than prior to dataset splitting. Continuous variables were imputed with the median, and categorical variables with the mode. This approach preserved the sample size for robust model training while acknowledging the potential for minor bias, which is discussed in the limitations.

### 2.2 Automated machine learning model development

Patients were randomly allocated into training (*n* = 671, 70%) and independent test (*n* = 287, 30%) sets. We developed an AutoML framework predicated on the Improved Hannibal Barcid Optimizer (IHBO) to simultaneously optimize feature selection and hyperparameter tuning for CRO prediction. The Hannibal Barcid Optimizer (HBO) algorithm translates military strategic principles—specifically the triple-enrollment tactics of the Battle of Cannae, historical frontline-based trend perception, and a parallax learning mechanism balancing tactical surprise with exploration—into computational optimization procedures. The IHBO enhancement incorporated chaotic mapping to improve initial population distribution ergodicity and a dynamic Lévy flight strategy to regulate exploration-exploitation tradeoffs during iterative search, with algorithmic validation against CEC2022 benchmark functions (Supplementary Figure S1).

**FIGURE 1:**
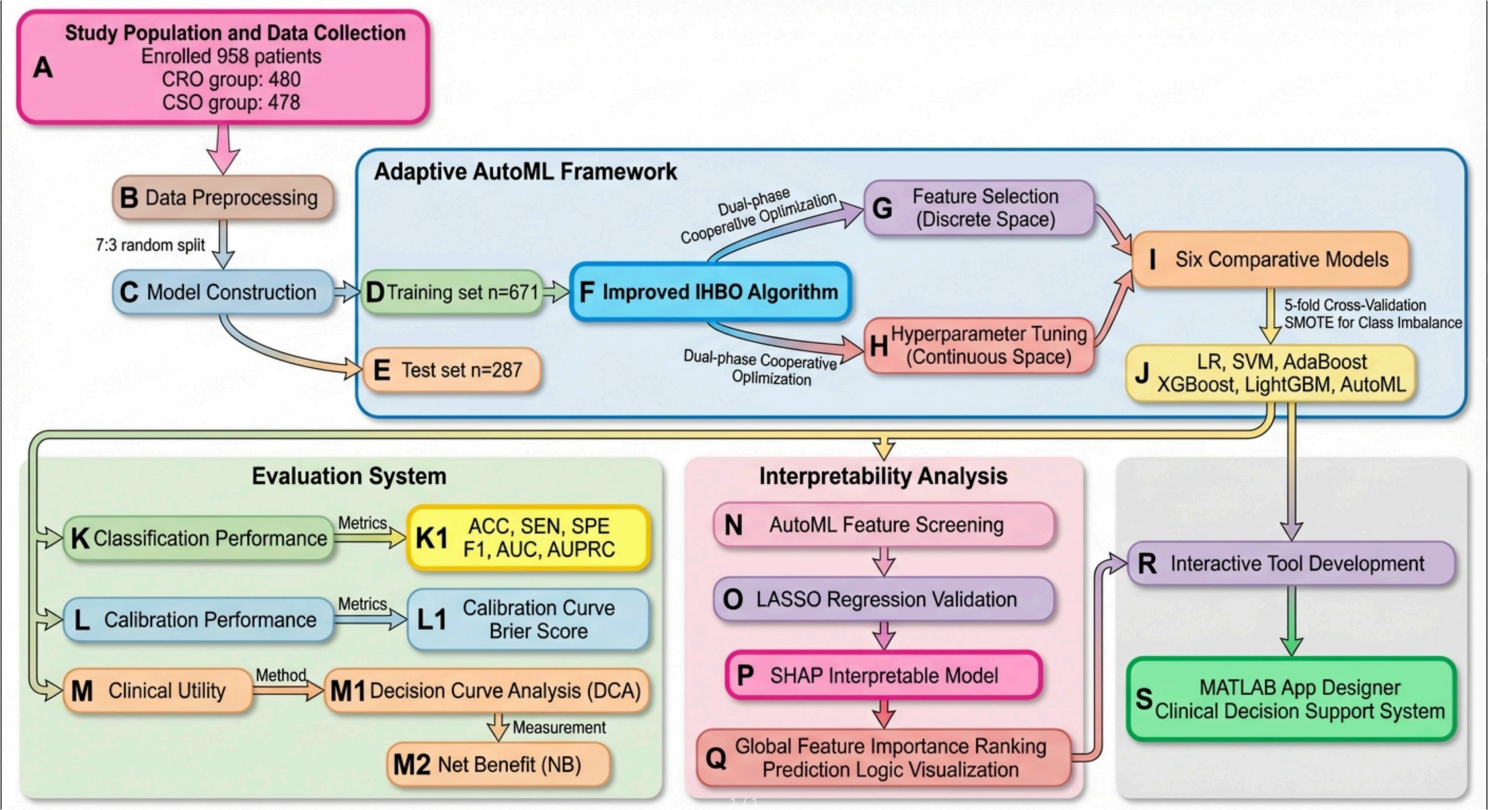
Flow chart of the study.

The IHBO algorithm drives a two-stage optimization process within the AutoML framework. Stage 1 (Feature Selection in Discrete Space): Each candidate solution (agent) in the IHBO population encodes a binary feature mask *F* = [f1, f2,…, fd], where fi ∈ {0,1} and d is the total number of initial features. fi = 1 indicates the feature is selected for model building, while fi = 0 indicates it is discarded. The IHBO optimizes this binary string by evolving the population based on fitness, which is the performance (F1-score) of a model trained using only the selected features. This is an embedded feature selection method, where selection is integral to the model training process. No pre-defined importance score threshold is used; the optimization directly seeks the feature subset that maximizes predictive performance. Stage 2 (Hyperparameter Tuning in Continuous Space): Concurrently, each agent also encodes a set of real-valued hyperparameters ^Θ^ for the base learner (e.g., learning rate, tree depth for XGBoost). IHBO optimizes these continuous parameters to fine-tune the model built on the feature subset from Stage 1. The two stages are co-optimized: for a given feature mask F, IHBO evaluates the fitness by training a model with hyperparameters ^Θ^ on the training data projected onto the selected features. This synchronous optimization ensures that feature selection and model configuration are jointly tailored for optimal prediction. The final output is the best-performing agent, providing the optimal feature subset and the corresponding tuned hyperparameters. Six benchmark models—Logistic Regression (LR), Support Vector Machine (SVM), AdaBoost, XGBoost, LightGBM, and our AutoML framework—were implemented in MATLAB 2024b. The model performed five-fold cross-validation on the training set. Figure 1 delineates the comprehensive analytical workflow.

### 2.3 Evaluation metrics

A multidimensional evaluation system was established: (1) Classification Performance: Accuracy (ACC), Sensitivity (SEN), Specificity (SPE), Precision (PRE), F1-score (F1), area under the receiver operating characteristic curve (ROC-AUC), and area under the precision-recall curve (PR-AUC) systematically assessed discriminative ability and stability under class imbalance. (2) Calibration Performance: Calibration curves and Brier score (lower values indicate higher accuracy) evaluated probabilistic prediction calibration. (3) Clinical Application: Decision Curve Analysis (DCA) quantified clinical utility by calculating net benefit (NB) across risk thresholds (pt):


$$N\,B=\frac{T\,P}{N}-\frac{F\,P}{N}\times \frac{p_{t}}{1-p_{t}}$$

(Where *TP*: true positives, *FP*: false positives, *N*: total samples). *NB* was compared against traditional intervention benchmarks to determine effective decision thresholds.

### 2.4 Feature selection, validation, and interpretability workflow

A three-step analytical workflow was adopted to ensure robust and interpretable feature selection: (1) Preliminary feature screening via IHBO-AutoML: The IHBO-AutoML framework performed embedded feature selection (as described in section 2.2) as part of its optimization process. By directly optimizing model performance, it identified the most predictive subset from the full feature set, yielding an initial candidate predictor set. (2) Robustness validation using LASSO regression: To verify the stability and sparsity of AutoML-selected features while preventing overfitting, Least Absolute Shrinkage and Selection Operator (LASSO) regression with 10-fold cross-validation was applied to the training set. This filtering method served as independent validation. (3) Interpretability analysis using SHAP: The final prediction model (IHBO-AutoML) built upon Step 1 features was interpreted using SHapley Additive exPlanations (SHAP). SHAP values quantified each feature’s contribution to individual predictions, providing global importance rankings and insights into local decision logic and feature interactions. This step translated model predictions into clinically comprehensible terms.

The final model deployed in clinical tools utilized five key features identified and optimized by the IHBO-AutoML framework. The LASSO analysis served as validation, demonstrating alignment between automated and traditional statistical selection methods regarding clinically plausible drivers of CRO risk.

### 2.5 Interactive tool development

MATLAB App Designer was deployed to build a clinical decision support software. This tool integrates the prediction model, providing clinicians with an intuitive interface for transfusion risk assessment. The system is web-deployable for clinical implementation.

### 2.6 Statistical methods

Data were analyzed in SPSS v26.0. Continuous variables were expressed as mean ± standard deviation (x̄ ± s) if normally distributed or median [interquartile range (IQR)] otherwise. Categorical variables were reported as frequencies and percentages [n (%)]. Intergroup comparisons used independent *t*-tests (normally distributed data), Mann-Whitney U tests (non-normal data), or Pearson’s χ^2^ tests (categorical data). Significance was set at α = 0.05 (two-tailed), with results presented in structured tables.

## 3 Results

### 3.1 Characteristics of the study population

The final cohort comprised 958 patients, with 480 in the Carbapenem-resistant Organisms (CRO) group and 478 in the Carbapenem-susceptible Organisms (CSO) group. The mean age was 60.93 ± 14.07 years, and 62.00% (*n* = 594) were male. Random allocation into training (*n* = 671) and testing (*n* = 287) sets demonstrated comparable baseline characteristics (all *P* > 0.05), with nearly identical CRO proportions between sets (training: 50.07% vs. testing: 50.17%; χ^2^ = 0.001, *P* = 0.977), confirming effective stratification. Detailed demographic and clinical profiles are presented in Table 1.

**TABLE 1:** Comparison of clinical characteristics between training set and testing set.

Feature	Training set (*n* = 671)	Testing set (*n* = 287)	Statistic	*P*-value
Outcome measure				
CRO infection, n(%)	336 (50.07)	144 (50.17)	0.001	0.977
Demographics				
Age > 60 years, n(%)	366 (54.55)	157 (54.70)	0.002	0.964
Male, n(%)	412 (61.40)	182 (63.41)	0.346	0.556
Comorbidities				
Hypertension, n(%)	256 (38.15)	112 (39.02)	0.065	0.799
Diabetes mellitus, n(%)	178 (26.53)	81 (28.22)	0.293	0.588
Coronary artery disease, n(%)	152 (22.65)	70 (24.39)	0.341	0.559
Cerebrovascular disease, n(%)	185 (27.57)	77 (26.83)	0.056	0.814
Chronic kidney disease, n(%)	87 (12.97)	35 (12.20)	0.107	0.743
Malignancy, n(%)	201 (29.96)	91 (31.71)	0.291	0.589
Immunosuppression, n(%)	72 (10.73)	30 (10.45)	0.016	0.899
Infection and Treatment Factors				
Sputum specimen source, n(%)	590 (87.93)	252 (87.80)	0.003	0.957
Hospitalization > 20 days, n(%)	301 (44.86)	129 (44.95)	0.001	0.980
ICU admission, n(%)	402 (59.91)	176 (61.32)	0.168	0.682
Stratified ICU length of stay, n(%)			0.238	0.888
0 days	281 (41.88)	124 (43.21)		
1–7 days	185 (27.57)	75 (26.13)		
> 7 days	205 (30.55)	88 (30.66)		
Surgical history, n(%)	295 (43.96)	124 (43.21)	0.047	0.828
Mechanical ventilation, n(%)	268 (39.94)	121 (42.16)	0.411	0.522
Central venous catheterization, n(%)	315 (46.95)	138 (48.08)	0.105	0.746
Indwelling urinary catheter, n(%)	387 (57.68)	169 (58.89)	0.121	0.728
Arterial catheterization, n(%)	105 (15.65)	47 (16.38)	0.080	0.778
Vasoactive medication use, n(%)	166 (24.74)	73 (25.44)	0.052	0.820
Antimicrobial Exposure				
Antimicrobial therapy > 7 days, n(%)	554 (82.56)	237 (82.58)	< 0.001	0.996
Carbapenems, n(%)	254 (37.85)	112 (39.02)	0.117	0.733
Aminoglycosides, n(%)	198 (29.51)	88 (30.66)	0.128	0.721
Glycopeptides, n(%)	156 (23.25)	65 (22.65)	0.041	0.840
Fluoroquinolones, n(%)	223 (33.23)	91 (31.71)	0.213	0.645
Third-generation cephalosporins, n(%)	374 (55.74)	162 (56.45)	0.041	0.840
β-lactam/β-lactamase inhibitor combinations, n(%)	450 (67.06)	196 (68.29)	0.138	0.710
Penicillins, n(%)	169 (25.19)	70 (24.39)	0.068	0.794
Antifungal agents, n(%)	145 (21.61)	65 (22.65)	0.127	0.722
Disease Severity Scores				
qSOFA score ≥ 2, n(%)	185 (27.57)	79 (27.53)	< 0.001	0.989
Charlson Comorbidity Index (CCI), M[Q1, Q3]	2.0 [1.0, 3.0]	2.0 [1.0, 3.0]	0.215	0.830
APACHE II score, M[Q1, Q3]	18.0 [12.0, 25.0]	18.0 [12.0, 24.0]	0.328	0.743
APACHE II score > 15, n(%)	201 (29.96)	88 (30.66)	0.048	0.827

Compared with the CSO group, the CRO group exhibited significantly higher proportions of age > 60 years, hypertension, diabetes mellitus, hospitalization > 20 days, ICU admission, ICU stay > 7 days, mechanical ventilation, central venous catheterization, indwelling urinary catheter, carbapenem use, aminoglycoside use, β-lactam/β-lactamase inhibitor combination use, antifungal agent use, qSOFA ≥ 2, and APACHE II > 15. Additionally, the CRO group demonstrated higher APACHE II scores (22.0 vs. 14.0). All these differences were statistically significant (*P* < 0.05). No statistically significant differences (*P* > 0.05) were observed between the two groups in terms of gender, Charlson Comorbidity Index (CCI), certain comorbidities, or treatment factors (see Table 2).

**TABLE 2:** The baseline characteristics between CRO and CSO patients.

Feature	CRO (*n* = 480)	CSO (*n* = 478)	Statistic	*P*-value
Demographics				
Age > 60 years, n(%)	314 (65.42)	209 (43.72)	45.465	< 0.001
Male, n(%)	291 (60.62)	303 (63.39)	0.777	0.378
Comorbidities				
Hypertension, n(%)	221 (46.04)	147 (30.75)	23.663	< 0.001
Diabetes mellitus, n(%)	155 (32.29)	104 (21.76)	13.473	< 0.001
Coronary artery disease, n(%)	107 (22.29)	115 (24.06)	0.420	0.517
Cerebrovascular disease, n(%)	128 (26.67)	134 (28.03)	0.225	0.635
Chronic kidney disease, n(%)	62 (12.92)	60 (12.55)	0.029	0.866
Malignancy, n(%)	152 (31.67)	140 (29.29)	0.639	0.424
Immunosuppression, n(%)	49 (10.21)	53 (11.09)	0.195	0.659
Infection and treatment factors				
Sputum specimen source, n(%)	418 (87.08)	424 (88.70)	0.590	0.442
Hospitalization > 20 days, n(%)	258 (53.75)	172 (35.98)	30.560	< 0.001
ICU admission, n(%)	347 (72.29)	231 (48.33)	57.476	< 0.001
Surgical history, n(%)	201 (41.88)	218 (45.61)	1.355	0.244
Mechanical ventilation, n(%)	233 (48.54)	156 (32.64)	25.123	< 0.001
Central venous catheterization, n(%)	272 (56.67)	181 (37.87)	33.961	< 0.001
Indwelling urinary catheter, n(%)	361 (75.21)	195 (40.79)	116.463	< 0.001
Arterial catheterization, n(%)	73 (15.21)	79 (16.53)	0.312	0.576
Vasoactive medication use, n(%)	117 (24.38)	122 (25.52)	0.169	0.681
Stratified ICU length of stay, n(%)				
0 days	140 (29.17)	265 (55.44)	70.563	< 0.001
1–7 days	150 (31.25)	110 (23.01)		
> 7 days	190 (39.58)	103 (21.55)		
Antimicrobial exposure				
Antimicrobial therapy > 7 days, n(%)	425 (88.54)	366 (76.57)	23.852	< 0.001
Carbapenems, n(%)	220 (45.83)	146 (30.54)	23.714	< 0.001
Aminoglycosides, n(%)	186 (38.75)	100 (20.92)	36.356	< 0.001
Glycopeptides, n(%)	108 (22.50)	113 (23.64)	0.175	0.675
Fluoroquinolones, n(%)	160 (33.33)	154 (32.22)	0.135	0.713
Third-generation cephalosporins, n(%)	279 (58.13)	257 (53.77)	1.847	0.174
β-lactam/β-lactamase inhibitor combinations, n(%)	388 (80.83)	258 (53.97)	78.670	< 0.001
Penicillins, n(%)	117 (24.38)	122 (25.52)	0.169	0.681
Antifungal agents, n(%)	136 (28.33)	74 (15.48)	23.114	< 0.001
Disease severity scores				
qSOFA score ≥ 2, n(%)	158 (32.92)	106 (22.18)	13.841	< 0.001
APACHE II score > 15, n(%)	184 (38.33)	105 (21.97)	30.454	< 0.001
Charlson Comorbidity Index (CCI), M [Q1, Q3]	2.0 [1.0, 3.0]	2.0 [1.0, 3.0]	1.048	0.279
APACHE II score, M [Q1, Q3]	22.0 [19.0, 25.0]	14.0 [12.0, 17.0]	23.610	< 0.001

### 3.2 Model training and validation

**FIGURE 2:**
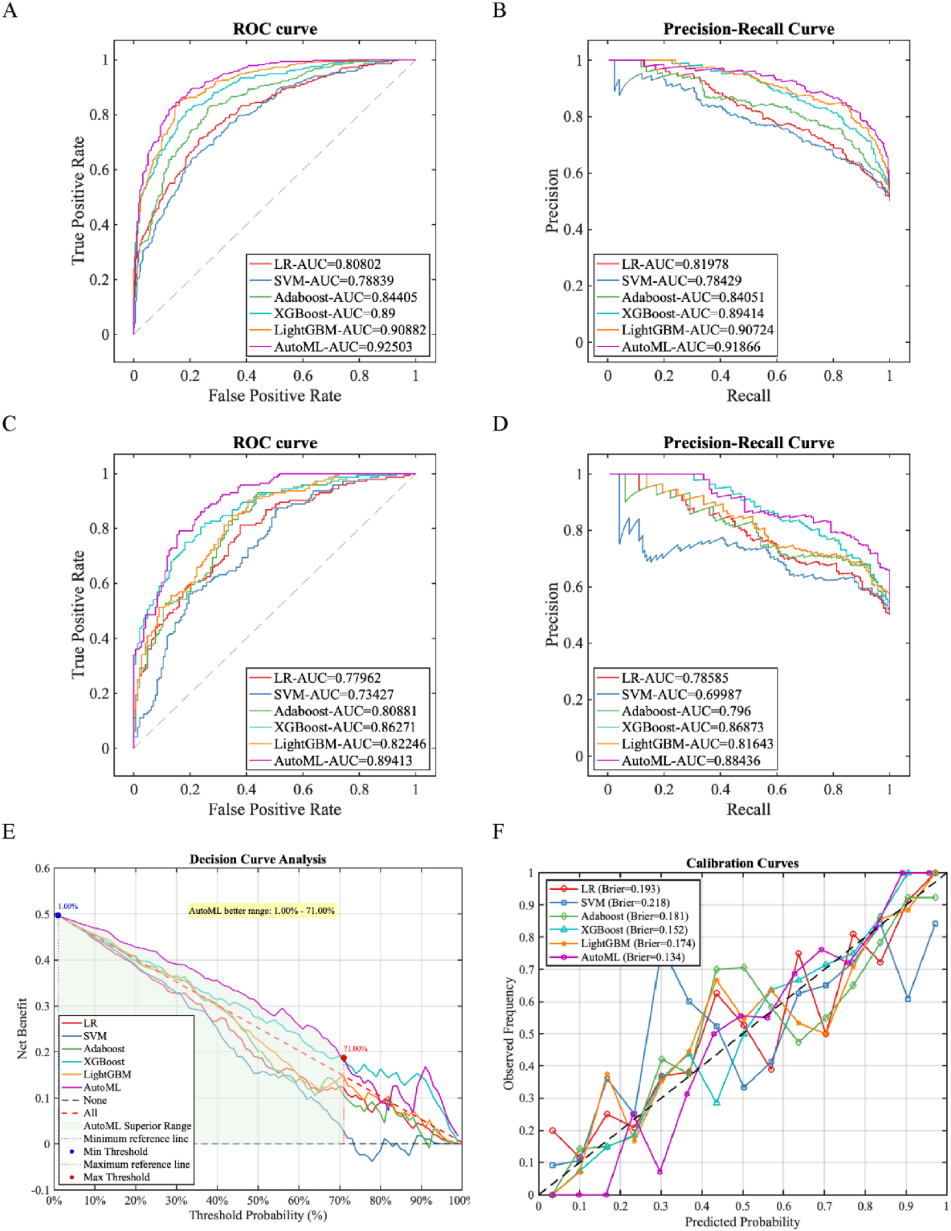
Model training and validation performance. This figure comprehensively evaluates the model’s performance on both the training set and an independent testing set. **(A,B)** The Receiver Operating Characteristic (ROC) curve and the Precision-Recall (PR) curve on the training set, respectively. Their x-axes represent the False Positive Rate and Recall, while the y-axes represent the True Positive Rate and Precision, with different curves illustrating the performance of different models. **(C,D)** Correspondingly display the ROC and PR curves on the independent testing set, assessing the model’s generalization capability. **(E)** The Decision Curve Analysis on the testing set, with the x-axis representing the threshold probability, the y-axis representing the standardized net benefit. Each curve depicts the clinical utility of different decision strategies, compared against the “treat all” and “treat none” extreme strategies. **(F)** The calibration curve on the testing set, where the x-axis is the mean predicted risk by the model, and the y-axis is the observed actual event rate. Scatter points with error bars indicate data binning, and the diagonal dashed line represents the ideal scenario of perfect agreement between predicted and observed risk.

Internal validation results on the training set revealed that all six compared prediction models demonstrated discernible discriminative capability (Figures 2A,B and Table 3). Among them, the AutoML model achieved the optimal comprehensive performance (ROC-AUC: 0.9250). Its high sensitivity (0.8452) and specificity (0.8507) indicated effectiveness in identifying high-risk patients while minimizing excessive alerts for low-risk individuals. This balanced performance holds significant clinical value for implementing precision interventions under resource-constrained conditions. External validation on the independent test set assessed model generalizability (Figures 2C,D and Table 3). The AutoML model maintained superior discriminative ability (ROC-AUC: 0.8941), with its outstanding specificity (0.8392) and precision (0.8321) signifying high predictive reliability when classifying patients as high-risk. This reliability empowers clinicians to confidently initiate or escalate preventive measures—such as advance microbiological testing or contact precautions—potentially reducing empirical carbapenem misuse and containing resistant pathogen transmission. Although its test-set sensitivity (0.7917) was lower than some models, this calibrated balance between sensitivity and specificity demonstrates greater clinical utility and operational feasibility than models sacrificing specificity for extreme sensitivity. It achieves an optimal trade-off between effective screening and avoidance of unnecessary medical burden.

**TABLE 3:** Performance metrics of predictive models.

Data set	Models	PRE	SEN	SPE	ACC	F1	ROC-AUC	PR-AUC
Training set	LR	0.6863	0.8333	0.6179	0.7258	0.7527	0.8080	0.8198
	SVM	0.6837	0.7976	0.6299	0.7139	0.7363	0.7884	0.7843
	Adaboost	0.7561	0.8304	0.7313	0.7809	0.7915	0.8441	0.8405
	XGBoost	0.8149	0.8125	0.8149	0.8137	0.8137	0.8900	0.8941
	LightGBM	0.8412	0.8512	0.8388	0.8450	0.8462	0.9088	0.9072
	AutoML	0.8503	0.8452	0.8507	0.8480	0.8478	0.9250	0.9187
Testing set	LR	0.6842	0.8125	0.6224	0.7178	0.7429	0.7796	0.7859
	SVM	0.6332	0.8750	0.4895	0.6829	0.7347	0.7343	0.6999
	Adaboost	0.6802	0.9306	0.5594	0.7456	0.7859	0.8088	0.7960
	XGBoost	0.7662	0.8194	0.7483	0.7840	0.7919	0.8627	0.8687
	LightGBM	0.6915	0.9028	0.5944	0.7491	0.7831	0.8225	0.8164
	AutoML	0.8321	0.7917	0.8392	0.8153	0.8114	0.8941	0.8844

Decision curve analysis (Figure 2E) demonstrated superior clinical net benefit across 1–71% threshold probabilities, indicating strong generalizability. Calibration curves (Figure 2F) confirmed enhanced reliability (Brier score = 0.134), substantially outperforming comparators.

### 3.3 Analysis of critical risk factors

#### 3.3.1 LASSO regression

**FIGURE 3:**
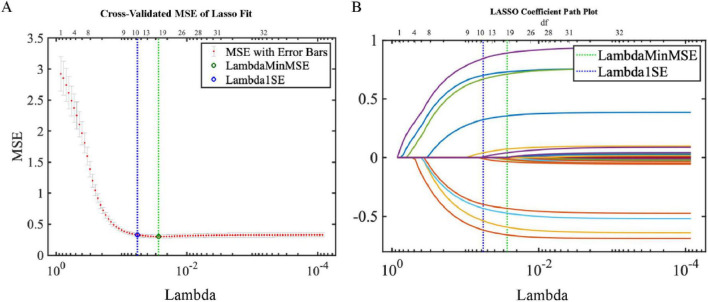
LASSO regression results. **(A)** Coefficient trajectories **(B)** Cross-validation with minimum MSE.

Feature selection via LASSO regression (Lambda1SE criteria) identified 11 significant variables (Figure 3). Crucially, all features selected by AutoML were included, validating our feature engineering pipeline.

#### 3.3.2 SHAP interpretation

**FIGURE 4:**
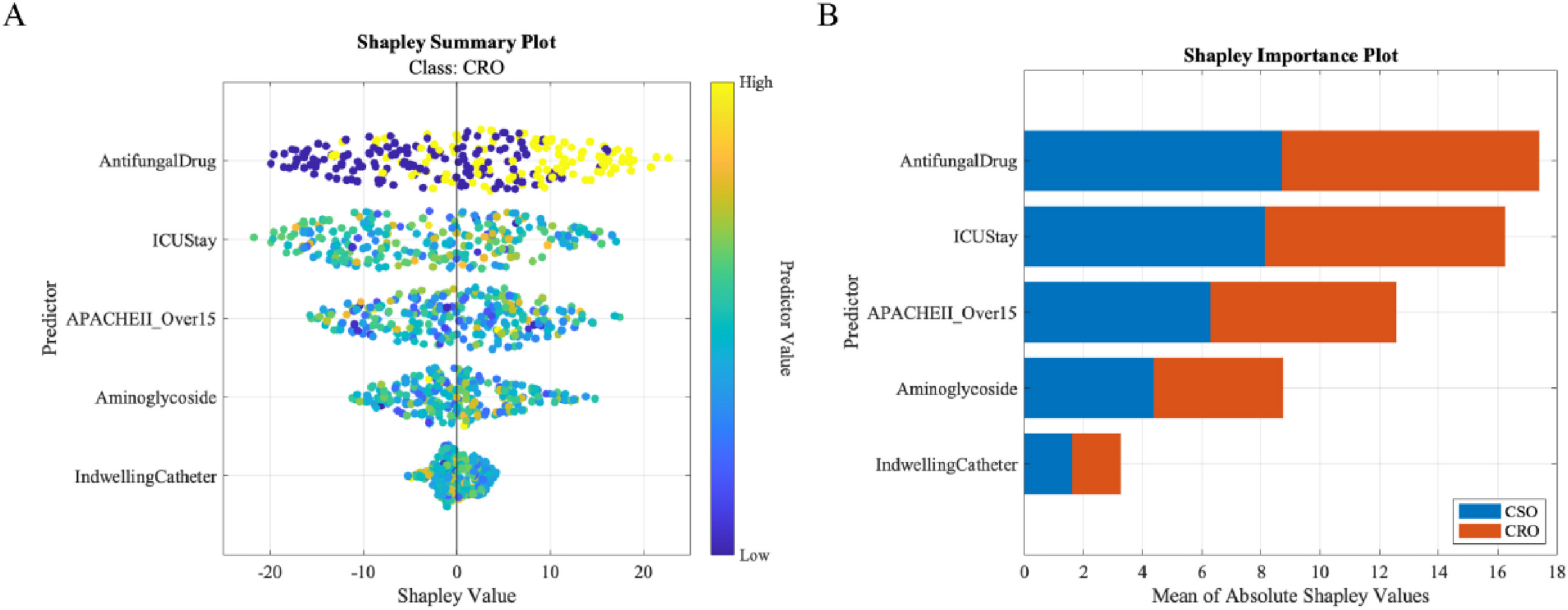
Global SHAP analysis. **(A)** Summary plot. **(B)** Feature importance ranking.

**FIGURE 5:**
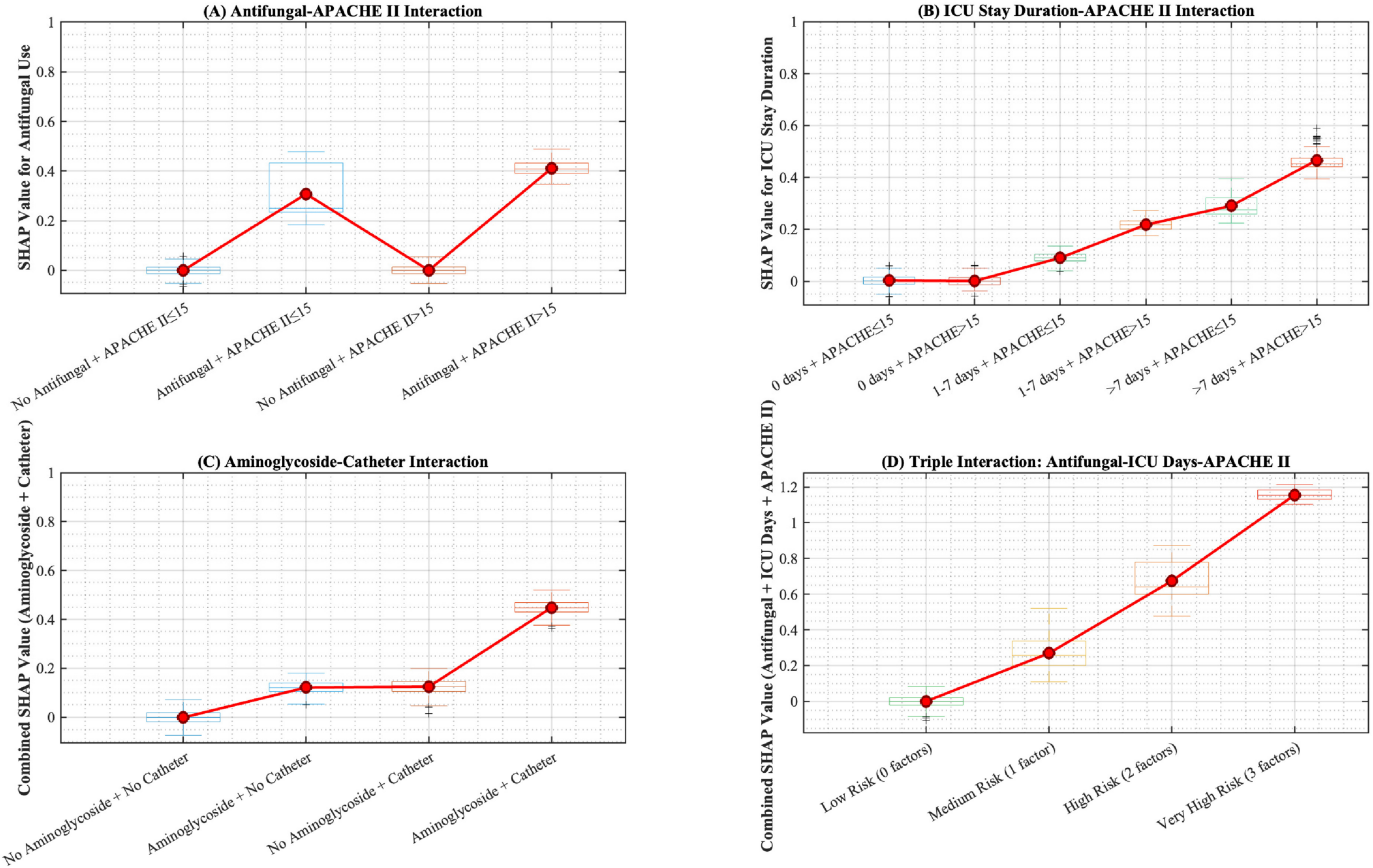
SHAP interaction effects. **(A–C)** Pairwise feature interactions. **(D)** High-risk triad synergy.

Global SHAP analysis (Figure 4) revealed top predictors: antifungal use, ICU duration, APACHE II > 15, aminoglycoside exposure, and indwelling catheters. Interaction analysis (Figure 5) elucidated critical synergies: (A) Antifungal use significantly increases CRO risk, especially in patients with APACHE II > 15 (red line shows elevated SHAP values in high-risk group); (B) ICU stay duration shows dose-response relationship with CRO risk, amplified when APACHE II > 15 (longer ICU stay with high APACHE II shows highest risk); (C) Combined use of aminoglycoside and urinary catheter creates synergistic risk (red diamonds in group 4), indicating additive effect of multiple risk factors; (D) Triple high-risk combination (antifungal + ICU > 7 days + APACHE II > 15) shows highest SHAP values, requiring intensive infection control measures.

(3) Contrast Between LASSO and AutoML in Feature Selection

LASSO regression identified 11 features with non-zero coefficients using L1 regularization, constructing a multidimensional predictive model spanning demographics, comorbidities, treatment intensity, and disease severity. In sharp contrast, the IHBO-optimized AutoML framework selected a highly streamlined core set of only 5 top predictors. Crucially, all 5 features—antifungal use, ICU length of stay, APACHE II score > 15, aminoglycoside exposure, and indwelling urinary catheter—were encompassed within LASSO’s 11-feature set, indicating strong consensus on core prognostic drivers. LASSO uniquely incorporated six additional features: age > 60, hypertension, diabetes, hospital stay > 20 days, mechanical ventilation, and carbapenem use (Table 4). Collectively, feature selection confirms that variables reflecting critical care intensity, invasive procedures, complex antibiotic regimens, and objective severity scoring are pivotal for predicting adverse outcomes in nosocomial infections. The principal distinction lies in model complexity and focus—AutoML prioritizes parsimony, while LASSO captures broader clinical dimensions.

**TABLE 4:** Feature comparison between AutoML and LASSO models.

Feature	AutoML SHAP	LASSO β -coefficient	Overlap
Antifungal Use	8.70	0.08	Yes
ICU Length of Stay	8.13	0.14	Yes
APACHE II score > 15	6.29	0.20	Yes
Aminoglycoside exposure	4.38	0.05	Yes
Indwelling urinary catheter	1.63	0.11	Yes
Age > 60	N/A	0.16	No
Hypertension	N/A	0.07	No
diabetes	N/A	0.09	No
Hospital stay > 20 days	N/A	0.12	No
Mechanical ventilation	N/A	0.10	No
Carbapenem use	N/A	0.06	No

#### 3.3.3 Clinical decision support tool

**FIGURE 6:**
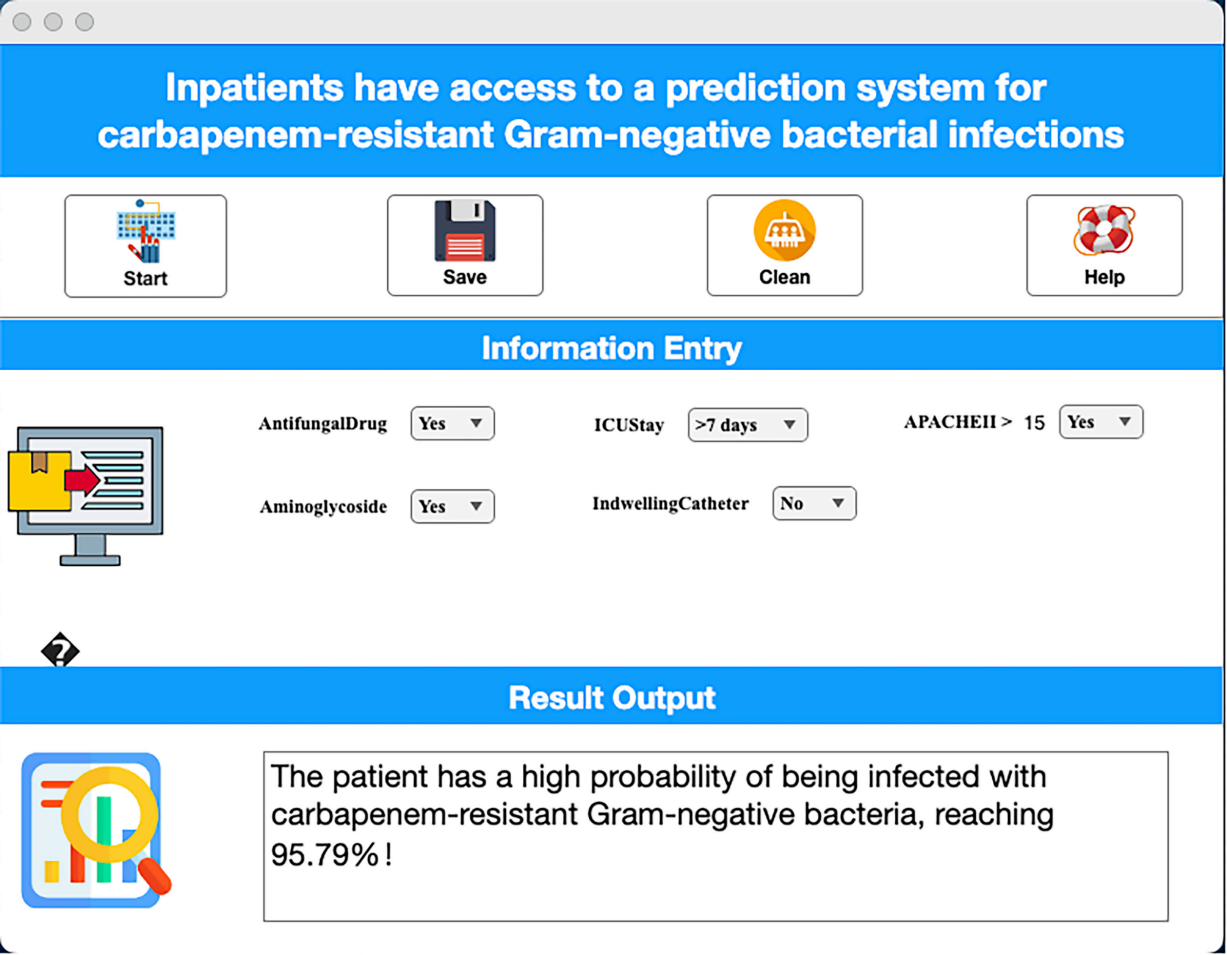
Clinical decision support interface demonstration.

To overcome implementation barriers requiring programming expertise, we developed an intuitive web-based system (Figure 6). Clinicians input five key features (identified in Section 3.4) to instantly visualize individualized CRO risk probabilities, enabling point-of-care intervention.

## 4 Discussion

This study establishes that the IHBO-optimized AutoML predictive framework achieves exceptional performance in identifying Carbapenem-resistant Organisms (CRO) infection risk among hospitalized patients. Critically, our IHBO-optimized AutoML framework predicts carbapenem resistance status among infected patients rather than infection occurrence per se, as reflected by the explicit CSO group definition (culture-confirmed susceptible infections). Consequently, the model is clinically positioned as a resistance stratification tool for diagnosed infections, enabling targeted antimicrobial stewardship through point-of-care resistance probability visualization. This distinction refines its utility to antimicrobial optimization scenarios rather than hospital-wide infection screening. Its predictive superiority stems not only from algorithmic innovations but also from the multidimensional interaction network revealed through SHAP analysis. The identification of antifungal therapy as a core predictor aligns with the ‘tripartite selection pressure’ hypothesis proposed by Todd et al., (2023). This suggests a potential mechanism involving antifungal agents reshaping gut microbiota ecology, inducing abnormal accumulation of fungal metabolites, compromising innate immunity, and creating ecological niches potentially conducive to horizontal resistance gene transfer (Meng et al., 2022; d’Enfert et al., 2021). Critically, this effect exhibits exponential amplification in severely ill patients, exposing a bidirectional positive feedback loop between immune dysfunction and microbial dysbiosis.

At the pathophysiological intersection, two interwoven pathways emerge: (i) On the immune axis, critical illness induces functional exhaustion of gut mucosal sentinel cells coupled with dysregulated immunoregulatory pathways, significantly impairing antifungal barrier defenses (Mellinghoff et al., 2022; Aggor et al., 2024); (ii) On the microbial axis, antifungal selection pressure drives adaptive expansion of resistance gene carriers within Enterobacteriaceae, accelerating interspecies transmission of resistance determinants (Sheng et al., 2021; Chen et al., 2024). The convergence of these biological processes culminates in irreversible multidrug-resistant pathogen proliferation. This theoretical insight delineates a transformable risk boundary: Concurrent antifungal exposure and critical illness beyond a threshold triggers discontinuous infection risk escalation, providing scientific justification for preemptive interventions that shift resistance management from reactive to proactive paradigms. Targeted disruption of the immunity-microbiota vicious cycle during critical time windows opens new avenues for personalized infection prevention.

ICU length of stay (LoS) emerged as the second most critical predictor, with its dose-response relationship reflecting ecological selection pressures inherent to intensive care environments. The “ICU Ecological Stress Triad” model elucidates this time-dependent risk accumulation: Synergistic effects among environmental selection pressure, invasive procedure density, and broad-spectrum antimicrobial intensity confer competitive advantages to resistant flora during adaptive evolution, establishing irreversible niche replacement patterns (Flaws et al., 2024). Particularly when coexisting with organ failure, this exposure manifests potent bio-synergy: Critical illness-induced gut barrier dysfunction and sustained ICU interventions form a pathogenic loop wherein colonization both results from and exacerbates clinical deterioration (Woodworth et al., 2025).

The predictive significance of aminoglycosides—contrasting with conventional carbapenem-centric risk paradigms—is elucidated through SHAP-interaction evidence demonstrating additive effects when combined with indwelling urinary catheters (Cariou et al., 2023). This interaction substantiates the anatomical niche-specific resistance evolution hypothesis: within urinary catheter biofilm microenvironments, sustained aminoglycoside pressure promotes 16S rRNA methyltransferase-mediated resistance and facilitates plasmid conjugation hotspots for carbapenemase gene transfer (Bull et al., 2025). Crucially, this ecodynamic mechanism is now corroborated by recent research on in vivo co-resistance selection in catheter-associated biofilms (Tamma et al., 2023). This device-drug interplay necessitates recalibrating resistance control from single-drug management toward anatomically-based ecological frameworks. The ICU Loos-organ failure synergy fundamentally exposes an ecological paradox in modern critical care: Life-support technologies inadvertently create spatiotemporal incubators for resistance evolution. This demands theoretical transformation from pharmacocentric control toward holistic medical ecosystem regulation, dynamically balancing therapeutic intensity with ecological preservation to sustain life support while disrupting resistance continuity—ultimately achieving dialectical unity between treatment efficacy and resistance containment.

Methodologically, the IHBO framework addresses two data modeling constraints: (i) Chaotic mapping initialization enhances feature space traversal with limited samples, enabling 11-core-variable retention while achieving PR-AUC 0.8844; (ii) Dynamic Lévy flight modulates exploration-exploitation balance, minimizing overfitting (Brier score = 0.134). Compared with Escudero-Arnanz’s model (Escudero-Arnanz et al., 2025), this framework reduces false positives while maintaining sensitivity—a critical advantage for preventing antimicrobial overuse. The interactive decision support tool transforms SHAP interaction analyses into actionable prevention pathways through its predictive-interventional closed-loop design. This overcomes the “alert without intervention” limitation of conventional models, providing a technical vehicle for individualized precision prevention. LASSO, as a generalized linear model, reflects the relatively independent linear contributions of each variable to prognosis. Meanwhile, the AutoML framework demonstrates remarkable predictive power in extracting synergistic information from these five highly correlated clinical variables, achieved through its robust nonlinear fitting and capability to capture feature interactions. Consequently, it discards other features that remain statistically significant in univariate or linear contexts. This implies that in real-world clinical environments with complex interactions, the impact of factors such as advanced age and specific comorbidities on prognosis might already be comprehensively represented or substituted by downstream decision indicators and status variables, such as “whether long-term ICU monitoring is required” and “whether the use of special-grade antimicrobial agents is necessary.” Although the LASSO model provides a richer variable set aiding in understanding prognostic risk profiles, the AI-optimized AutoML model identifies an exceptionally minimalistic feature subset composed almost entirely of high-intensity clinical interventions and objective scores. This subset not only demonstrates outstanding predictive performance but also, due to its direct association with clear clinical actions and assessment nodes, facilitates rapid risk evaluation and identification of high-risk patients in clinical practice, offering a novel perspective for precision medical decision-making. Future work will focus on validating the generalizability of this parsimonious model in external validation cohorts.

Several limitations warrant consideration and future research directions. Notably, the absence of external validation using independent multicenter datasets restricts the assessment of model generalizability across heterogeneous healthcare settings. Despite internal validity ensured by rigorous sampling and dual-stage feature selection, cross-center validation studies suggest that predictive models often exhibit significant performance degradation due to institutional variations, and region-specific healthcare resource heterogeneity may further constrain cross-institutional generalizability. Multicenter prospective validation through regional medical consortiums using standardized protocols is essential, integrating transfer learning to address data heterogeneity. Mechanistically, SHAP-identified antifungal drivers require multi-omics verification: Longitudinal metagenomic sequencing of fecal samples coupled with resistance gene quantification could elucidate fungal-bacterial interactions facilitating carbapenemase gene transfer; concurrent biofilm experiments should validate antifungal agents (e.g., fluconazol) in modulating resistance biofilm formation. Clinically, seamless integration requires three upgrades: FHIR-standardized hospital information system interfaces for dynamic risk dashboards; evidence-guided response modules (e.g., automated enhanced disinfection/rectal screening/antimicrobial stewardship consults for high-risk triads); and interrupted time series analysis evaluating impacts on CRO incidence and antimicrobial use intensity. Model simplification and health economic assessments remain vital for grassroots implementation.

## 5 Conclusion

This study successfully develops an IHBO-powered AutoML model for precise CRO infection risk stratification in hospitalized patients. Key contributions include: (i) SHAP-based elucidation of nonlinear interactions among five core predictors—notably synergistic effects between aminoglycosides and invasive procedures; and (ii) An innovative clinical decision support tool enabling closed-loop risk-to-intervention management. Beyond providing a technical template for proactive resistance containment, the feature interaction analysis framework offers methodological reference for complex medical prediction tasks such as sepsis early warning. Implementation research within regional healthcare alliances is recommended to evaluate model-guided prevention strategies’ health-economic impact.

## Data Availability

The raw data supporting the conclusions of this article will be made available by the authors, without undue reservation.
