# Supplementary material for: Development of a predictive model using automated machine learning for Carbapenem-resistant Organisms (CRO) infections in hospitalized patients
Source: Front Microbiol. 2026 Apr 1;17:1775344. doi: 10.3389/fmicb.2026.1775344 (PMC13079023; doi:10.3389/fmicb.2026.1775344)
Supplement: Supplementary file 1 [file Table_1.docx]

**Supplementary Materials**

**1.Missing value reporting and handling**

A missingness analysis revealed that all variables had a missing rate of <5%, with most key predictors (e.g., APACHE II score, antimicrobial exposure history) missing in <2% of cases. The detailed missing percentage for each variable is provided in Supplementary Table S1. For the minimal random missing data, single imputation was performed strictly before dataset splitting to prevent information leakage. Continuous variables were imputed with the median, and categorical variables with the mode. This approach preserved the sample size for robust model training while acknowledging the potential for minor bias, which is discussed in the limitations.

**Table S1. Completeness and Imputation of Key Variables in the Analytic Cohort (n=958)**

| **Category** | **Variable** | **Type** | **Missing, n** | **Missing, %** | **Imputation Method** |
| --- | --- | --- | --- | --- | --- |
| **Demographics & Basics** | Age | Continuous | 0 | 0.00% | - |
|  | Sex | Categorical | 0 | 0.00% | - |
|  | Body Mass Index (BMI) | Continuous | 36 | 3.76% | Median (22.8 kg/m²) |
| **Comorbidities** | Hypertension | Categorical | 0 | 0.00% | - |
|  | Diabetes mellitus | Categorical | 0 | 0.00% | - |
|  | Coronary artery disease | Categorical | 3 | 0.30% | Mode (No) |
|  | Cerebrovascular disease | Categorical | 2 | 0.20% | Mode (No) |
|  | Chronic kidney disease (Stage≥3) | Categorical | 5 | 0.50% | Mode (No) |
|  | Active Malignancy | Categorical | 0 | 0.00% | - |
|  | Immunosuppression* | Categorical | 7 | 0.70% | Mode (No) |
| **Admission Status & Source** | Admission Source (ED/Ward/Transfer) | Categorical | 0 | 0.00% | - |
|  | Primary Admission Department | Categorical | 0 | 0.00% | - |
| **Infection & Treatment Factors** | Specimen Source (Sputum/Blood/Urine/etc.) | Categorical | 0 | 0.00% | - |
|  | Hospitalization >20 days | Categorical | 0 | 0.00% | - |
|  | ICU admission | Categorical | 0 | 0.00% | - |
|  | Stratified ICU length of stay (0,1-7,>7 days) | Categorical | 0 | 0.00% | - |
|  | Surgical procedure (within 30 days) | Categorical | 0 | 0.00% | - |
|  | Mechanical ventilation (duration >48h) | Categorical | 4 | 0.40% | Mode (No) |
|  | Central venous catheterization | Categorical | 0 | 0.00% | - |
|  | Indwelling urinary catheter (duration >72h) | Categorical | 3 | 0.30% | Mode (No) |
|  | Arterial catheterization | Categorical | 8 | 0.80% | Mode (No) |
|  | Vasoactive medication use (≥24h) | Categorical | 0 | 0.00% | - |
| **Antimicrobial Exposure (>72h)** | Any antimicrobial therapy >7 days | Categorical | 0 | 0.00% | - |
|  | Carbapenems (e.g., Meropenem) | Categorical | 0 | 0.00% | - |
|  | Aminoglycosides (e.g., Amikacin) | Categorical | 0 | 0.00% | - |
|  | Glycopeptides (Vancomycin/Teicoplanin) | Categorical | 0 | 0.00% | - |
|  | Fluoroquinolones (e.g., Levofloxacin) | Categorical | 0 | 0.00% | - |
|  | Third-generation cephalosporins | Categorical | 0 | 0.00% | - |
|  | β-lactam/β-lactamase inhibitors (e.g., PIP/TAZ) | Categorical | 0 | 0.00% | - |
|  | Penicillins | Categorical | 0 | 0.00% | - |
|  | **Antifungal agents (e.g., Fluconazole)** | Categorical | 9 | 0.90% | Mode (No) |
| **Laboratory & Severity Scores** | White Blood Cell Count (peak, ×10⁹/L) | Continuous | 28 | 2.90% | Median (11.5) |
|  | Serum Albumin (lowest, g/L) | Continuous | 45 | 4.70% | Median (30.2) |
|  | Serum Creatinine (peak, μmol/L) | Continuous | 22 | 2.30% | Median (88.5) |
|  | qSOFA score ≥2 | Categorical | 0 | 0.00% | - |
|  | Charlson Comorbidity Index (CCI) | Continuous | 0 | 0.00% | - |
|  | **APACHE II score** | Continuous | 18 | 1.90% | Median (18.0) |
|  | APACHE II score >20 (derived) | Categorical | 18 | 1.90% | Imputed based on imputed APACHE II |
| **Outcome** | CRO Infection (Primary Outcome) | Categorical | 0 | 0.00% | - |

**2 Simulation Performance of Algorithm Improvement**

To evaluate the optimization capabilities of Improved Hannibal Barcid Optimizer (IHBO), comparative tests were conducted against original HBO and six benchmark algorithms (WOA, GWO, PSO, GA, GA-PSO, GA-ACO) using all 12 CEC2022 functions. Experiments with dimension=10, population=30, and maximum iterations=500 were independently replicated 30 times. Boxplots demonstrated IHBO's superior stability across most functions (Figure S1A). Convergence curve analysis further revealed IHBO's accelerated convergence rate and minimized local optima risk (Figure S2B), confirming significant advantages in global optimization efficiency.


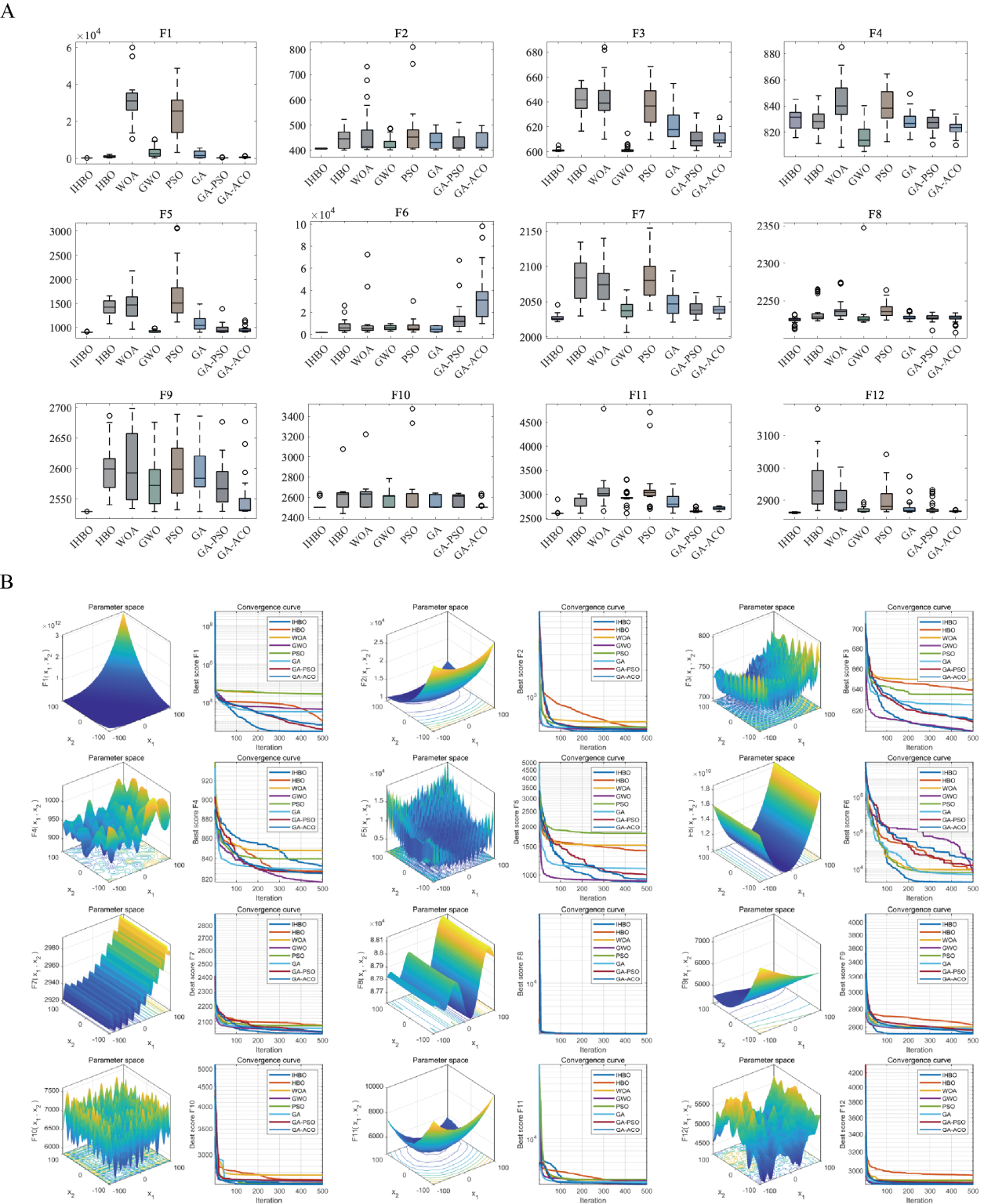


**Figure S1. Benchmark Testing of Improved Swarm Intelligence Algorithms**

Note: (A) Comparison of solution stability across different algorithms on each test function. This panel consists of 12 subplots corresponding to the 12 CEC2022 benchmark functions (F1–F12). In each subplot, the x-axis represents the eight algorithms compared (IHBO, HBO, WOA, GWO, PSO, GA, GA-PSO, GA-ACO), and the y-axis represents the best fitness value (optimal solution) obtained from 30 independent runs. The box plots illustrate the distribution and stability of the solutions obtained by each algorithm on a specific function. (B) Comparison of convergence curves across different algorithms on each test function. This panel consists of 12 subplots corresponding to the 12 CEC2022 benchmark functions (F1–F12). In each subplot, the x-axis represents the iteration number (1–500), and the y-axis represents the best fitness value of the current population (logarithmic scale). Each curve depicts the evolution of the best solution found by an algorithm as the iterations proceed. Experimental settings: All tests were conducted with a dimension of 10, a population size of 30, and a maximum of 500 iterations. The results shown are statistical summaries from 30 independent runs.
